# Availability and quality of paraffin blocks identified in pathology archives: A multi-institutional study by the Shared Pathology Informatics Network (SPIN)

**DOI:** 10.1186/1471-2407-7-37

**Published:** 2007-02-28

**Authors:** Ashokkumar A Patel, Dilipkumar Gupta, David Seligson, Eyas M Hattab, Ulysses J Balis, Thomas M Ulbright, Isaac S Kohane, Jules J Berman, John R Gilbertson, Sarah Dry, Osvaldo Schirripa, Hong Yu, Michael J Becich, Anil V Parwani, the Shared Pathology Informatics Network

**Affiliations:** 1 Department of Pathology, Center for Pathology Informatics, Benedum Oncology Informatics Center, University of Pittsburgh, Pittsburgh, PA, USA; 2 Department of Pathology, University of California, Los Angeles, CA, USA; 3 Department of Pathology, Indiana University, Indianapolis, IN, USA; 4 Department of Pathology, Harvard University, Boston, MA, USA; 5 Cancer Diagnosis Program, National Cancer Institute, National Institutes of Health, Bethesda, MD, USA

## Abstract

**Background:**

Shared Pathology Informatics Network (SPIN) is a tissue resource initiative that utilizes clinical reports of the vast amount of paraffin-embedded tissues routinely stored by medical centers. SPIN has an informatics component (sending tissue-related queries to multiple institutions via the internet) and a service component (providing histopathologically annotated tissue specimens for medical research). This paper examines if tissue blocks, identified by localized computer searches at participating institutions, can be retrieved in adequate quantity and quality to support medical researchers.

**Methods:**

Four centers evaluated pathology reports (1990–2005) for common and rare tumors to determine the percentage of cases where suitable tissue blocks with tumor were available. Each site generated a list of 100 common tumor cases (25 cases each of breast adenocarcinoma, colonic adenocarcinoma, lung squamous carcinoma, and prostate adenocarcinoma) and 100 rare tumor cases (25 cases each of adrenal cortical carcinoma, gastro-intestinal stromal tumor [GIST], adenoid cystic carcinoma, and mycosis fungoides) using a combination of Tumor Registry, laboratory information system (LIS) and/or SPIN-related tools. Pathologists identified the slides/blocks with tumor and noted first 3 slides with largest tumor and availability of the corresponding block.

**Results:**

Common tumors cases (*n *= 400), the institutional retrieval rates (all blocks) were 83% (A), 95% (B), 80% (C), and 98% (D). Retrieval rate (tumor blocks) from all centers for common tumors was 73% with mean largest tumor size of 1.49 cm; retrieval (tumor blocks) was highest-lung (84%) and lowest-prostate (54%).

Rare tumors cases (n = 400), each institution's retrieval rates (all blocks) were 78% (A), 73% (B), 67% (C), and 84% (D). Retrieval rate (tumor blocks) from all centers for rare tumors was 66% with mean largest tumor size of 1.56 cm; retrieval (tumor blocks) was highest for GIST (72%) and lowest for adenoid cystic carcinoma (58%).

**Conclusion:**

Assessment shows availability and quality of archival tissue blocks that are retrievable and associated electronic data that can be of value for researchers. This study serves to compliment the data from which uniform use of the SPIN query tools by all four centers will be measured to assure and highlight the usefulness of archival material for obtaining tumor tissues for research.

## Background

Repositories of clinically annotated human tissue specimens are vitally important to biomedical researchers[1,2]. In the past, tissue repositories were created prospectively by collecting samples of pre-determined types of lesions excised during surgical procedures. Such repositories are very expensive to create and are usually difficult to manage after their initial funding period terminates without additional resources. The Shared Pathology Informatics Network (SPIN) is a National Cancer Institution (NCI)-funded multi-institutional initiative designed to utilize the vast amounts of routinely stored paraffin-embedded tissue blocks as a ready-made tissue repository that can be automatically searched for blocks and data appropriate for many kinds of research efforts[3].

There are large collections of archived paraffin tissue already in existence for which many pathology laboratories have at least ten years of pathology reports stored electronically[4]. It is important to emphasis that these archived tissues are collected and stored at the time of routine diagnostic pathology services, whereas, most tissue bank collections are made up of targeted specimens that are specific to an organ system or tumor of interest. Searchable databases with clinical data on patients also exist at hospitals and medical institutions. Consequently, as part of the SPIN feasibility study, hospitals affiliated with Harvard University, Indiana University, University of California Los Angeles, and the University of Pittsburgh that make up the SPIN have developed a tool that is a working, freely distributable prototype for identification of available tissue specimens across nationally distributed tissue archives[5]. Prior to a full-scale implementation of the tool at the study sites, a feasibility study was necessary to determine whether or not the available specimens retrievable were of significant quality for performing research studies.

It has been estimated that pathology laboratories process 50 million specimens every year and it is possible that institutions in the U.S. have warehoused more than 300 million specimens[6]. While formalin fixed, paraffin embedded tissues are remarkably resilient and permit long term storage and retrieval for research use[4,7,8], there is a paucity of information on the quality and availability of such tissues. In the great majority of medical centers, it is not a priority to determine what is the nature and accessibility of the paraffin archives. While there is a general opinion that the archive may be valuable, once a case is signed out little work is done to control inventory, optimize storage locations and perform general quality assurance and quality control (QA/QC).

The first five years of funding for the SPIN effort was a feasibility study to determine if the tissue data (from electronic surgical pathology reports) could be successfully queried and if the quality and numbers of tissue blocks and clinical/pathology annotation would be adequate for researchers. The purpose of the present study is to assess the availability and quality of paraffin blocks that are identified by whatever means are chosen locally (i.e. the SPIN system or other local laboratory information systems (LIS) computer searches) based on a sampling approach. In order for the SPIN tool to be successfully used, the tissues must not only be identified, but the research team must also be provided with a realistic estimate of the availability and quality of the tissue. The objectives of this study therefore were (1) to obtain a sample of pathology reports and determine the percentage of cases where an institution can find the associated glass slides and/or tissue blocks for which tumor is still available for research purposes and (2) to determine the availability and quality of paraffin blocks that are identified by each institute's optimal search procedure.

## Methods

### Case selection and sampling approach

The focus of this study was the retrieval rates of quality paraffin blocks with the presence of tumor material from surgical pathology reports containing a cancer diagnosis. Thus, the target sample of pathology reports was limited to cases where adequate excised specimens could be available and reported the presence of certain cancer diagnoses in two major categories. The criteria used for the *common tumor *category consisted of 100 cases of the most common epithelial tumors (25 each from breast adenocarcinoma, colonic adenocarcinoma, lung squamous carcinoma, and prostate adenocarcinoma) collected between 1990 and 2005. The *rare tumor *criteria consisted of 100 rare or uncommon tumors (25 cases each of adrenal cortical carcinoma, gastrointestinal stromal tumor [GIST], adenoid cystic carcinoma, and mycosis fungoides) collected between 1990 and 2005. The work for this study was performed under the auspices of University of Pittsburgh IRB #0304081 and associated IRBs at the partnering institutions (Harvard, Indiana University and UCLA).

Consensus about the final list of tumor types was reached by the SPIN investigators. In particular, the list of tumor types originally selected for the rare tumor cases were adrenal cortical carcinoma, gastrointestinal stromal tumor (GIST), choriocarcinoma and retinoblastoma. We had limited success with the initial search of these rare tumors because there was site-specific variability in terms of obtaining the required number of cases. Specifically, at institutes having cases triaged at hospitals targeted to specific patient populations, such as children's and women's hospitals, there was a paucity of data retrieval for those specialized cancer cases, thus the above revised list for rare tumors was used for this study.

The year 1990 was chosen as the initial time frame for case identification because of two primary reasons. First, the fact that most of the SPIN member institutions electronic pathology record system was implemented by the late 1980s. It was essential to have the electronic pathology reports to demonstrate that the SPIN tools for identifying cases can utilize them. Second, the authors wanted to demonstrate the value for potential researchers that may use the SPIN tools and its advantage when linked to other electronic databases by presenting the retrieval rates of blocks and slides that would have at least 5–10 years of follow up data in other medical records. A SPIN pathologist randomly selecting cases for each year for initial screening and verification in order to select the first 100 eligible cases in each major category accomplished this. Also, an extra five-year time frame was added to allow every site to increase its chances of finding adequate number of rare tumor cases.

Each of the four SPIN institutions (Harvard, Indiana University, UCLA and U Pitt) was expected to randomly identify cases for the two major categories by the most accessible and widely available tools locally. These included the surgical pathology laboratory information system or LIS, Tumor Registry or SPIN-related informatics tools. It is important to note that each institution has different databases and specimen query mechanisms to identify cases of interest by default, and as such, the different methods used to identify cases are part of the routine workflow and represent each site's optimal search procedure. For example, for identifying the common tumors, while other institutions used the aforementioned methods, Harvard utilized primarily the SPIN tools for case finding which identified the most current cases that were undemanding to retrieve from on-site archives rather than off-site storage facilities. Thus, this manuscript primarily highlights the retrievable rates of quality archived paraffin blocks with the presence of tumor for research studies and not the method by which blocks were identified.

### SPIN study data forms

All data were recorded on a pre-defined Excel worksheet that was distributed to each lead pathologist at the beginning of this study. The Excel data forms had three sections that were filled by the project coordinator, tissue banker/technician that retrieved the slides/blocks, and the pathologist that reviewed the slides/blocks for tumor. The form consisted of a dropdown list of pre-defined choices for each data cell within the three sections.

The Coordinator distributed the Excel data form with pre-filled data cells for the four tumor types in the two major categories. The tissue banker acted as an honest broker who noted information about hospital and institution affiliations for locating the slides/blocks as well as the associated surgical pathology accession number as identified via each institutions preferred methods.

### Specimen Retrieval

Once the final 100 cases in each major category were identified, all reports were printed for final verification. Tissue bankers and/or histotechnologists searched for slides and blocks in the archives files; accessing either the on-site or off-site (warehoused) case materials. If the case was missing, and there was a tracking tag inserted in the file, tissue bankers attempted to track it down from the investigator who had utilized the materials. If the case was missing and there was no tracking tag, then the search stopped with the assumption that the block was unavailable. If the case was found, the material was then brought to the laboratory where it was inventoried and the data entered into the SPIN study data forms. Specifically, the tissue bankers noted the number of slides/blocks reported on the pathology report, in addition to the number of slides/blocks that they were actually able to retrieve from the pathology archives. If any slides/blocks were not retrieved, a comment section was provided to document why they were missing or if they were currently being used for other studies/investigators. The cases were then transferred to a study pathologist for review, along with copies of the partially completed Excel data forms.

### Specimen Review

Cases were reviewed by multiple pathologists in accordance with the standardized review protocol, and tabulated into the study data forms. The pathologists initially reviewed all the retrieved slides associated with a case and noted the total number of available slides/blocks with the presence of tumor. The size in diameter of the tumor was determined in each slide and the first 3 slides with largest tumor size were noted. The availability of the corresponding blocks in these 3 slides was also noted. In cases where slides were missing but blocks were found, new slides were not re-cut but a visual examination of the blocks was noted. For quality assessment, in 15% of randomly selected cases, the pathologist matched the outlines of the tissue in the paraffin blocks to the glass slides to determine the amount of tissue remaining in the paraffin block. The amount of tissue present on the blocks was visually examined for adequacy.

A lead pathologist who reviewed the data for completeness reconsolidated the completed data files into one file. The Excel data template with its resultant data set was further processed by an anonymizer, which substituted a code number for the locally available surgical accession number. Final de-identified data were electronically forwarded to the Pittsburgh contingent of the SPIN consortium for final analysis.

## Results

### Retrieval of common tumors

**Figure 1:**
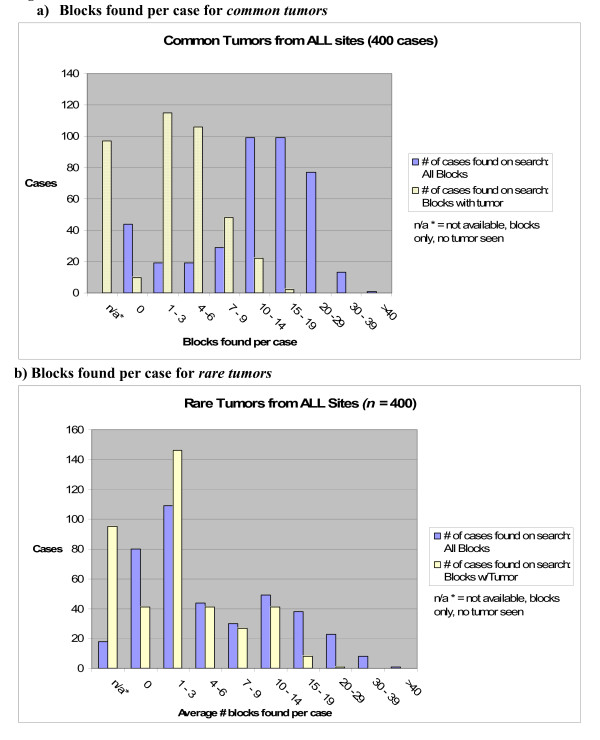
**Average blocks found per case from tissue archives**. Illustration of the availability of the cases found by all SPIN sites involving the search for (a) the *common tumor *type and (b) the *rare tumor *type categories. The breakdown of the average number of all blocks found per case after the initial search from the archives are compared to the number of blocks found with presence of tumor.

The summary of the available blocks and slides for the common tumor cases (*n *= 400) from the combined tissue archives of the four SPIN member institutions is shown in Table 1. Figure 1a illustrates the average number of paraffin embedded blocks/case (all blocks) that were available in the archives in parallel with the average number of tumor blocks/case that had been examined from all sites involving the search of common tumors. Of note, at least 73% of the cases found had between 1–14 blocks/case with tumor tissue available for potential research use.

For common tumors, the overall case retrieval was highest for lung (94%) and breast (93%), followed by colon (85%) and prostate (83%), as described in Table 2. Table 3 shows that each institution's case retrieval rates (all blocks) were 98% (UCLA), 95% (Indiana), 83% (Harvard), and 80% (Pitt), along with the percentage of cases with at least ≥1 block with presence of tumor tissue. Correspondingly, of the total blocks that were retrieved and examined from the archives, Table 4 shows the percentage of blocks with tumor present to be highest for lung (96%) and prostate (91%) followed by breast (88%) and colon (86%). UCLA (98%) and Indiana (95%) had the greatest percentage of blocks with tumor tissue found and are followed by Pittsburgh (85%) and Harvard (83%).

**Figure 2:**
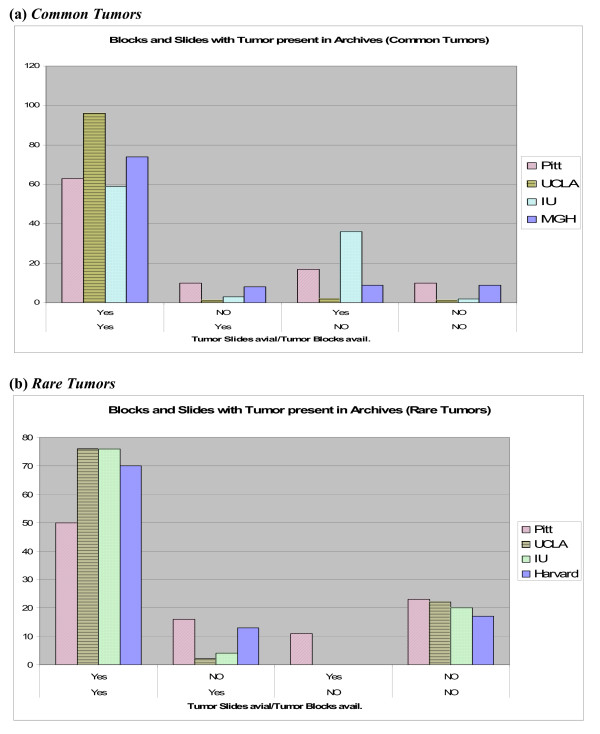
**Availability of slides and/or blocks with tumor**. Illustration of the availability of slides and/or blocks with the presence of tumor tissue from individual SPIN centers in (a) *common tumor *cases and (b) *rare tumor *cases.

**Figure 3:**
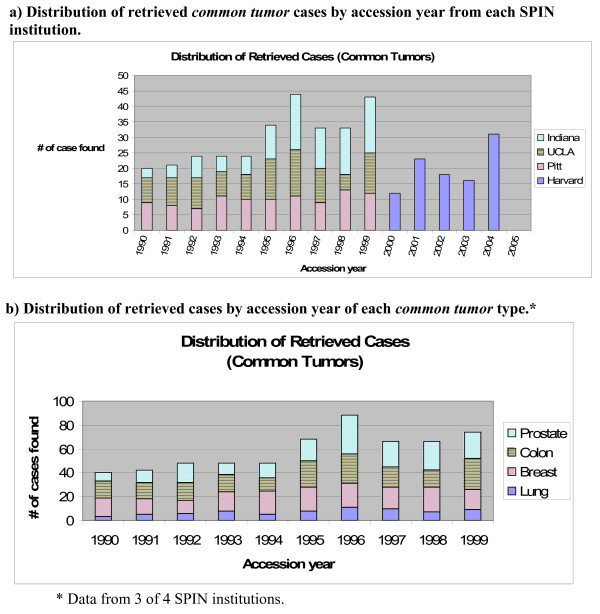
**Distribution of retrieved cases for the *common tumors***. Illustration of number of cases retrieved for common tumor cases by the original year of accession: (a) Breakdown by each institution and (b) by common tumor type*. Specimens retrieved and evaluated for the common tumors encompassed cases accessioned range from 1990–1999 with 64% being at least 10 years old.

Although many of the cases did have paired blocks and slides with tumor available, Figure 2a shows that there were 64 cases that had only slides with tumor available but no matching blocks were available; 22 cases had neither the slides nor the blocks. Retrieval rate for tumor blocks from all centers for common tumors was 73% with a mean largest tumor size of 1.49 cm, which is revealed in Table 5. The number of cases retrieved for the common tumor types by the original year of accession is shown in Figure 3a and 3b, showing the distribution across the SPIN institutions and across common tumor types, respectively. Specimens retrieved and evaluated for the common tumors encompassed cases accessioned from 1990–2005 with 64% being at least 10 years old.

### Retrieval of rare tumors

The summary of the available blocks and slides for the rare tumor cases (*n *= 400) from the tissue archives of the four SPIN member institutions is shown in Table 6. Figure 1b illustrates the average number of total blocks/case found for the rare tumor group in parallel with the range of blocks that showed presence of tumor tissue per case. Of significance is the fact that at least 64% of the cases found had between 1–14 blocks/case with tumor tissue available for potential research use.

Among the rare tumors, the overall case retrieval was highest for mycosis fungoides (83%) and GIST (82%) followed by adrenal cortical carcinoma (71%) and adenoid cystic carcinoma (66%), as described in Table 7. Table 8 shows that each institution's case retrieval rates (all blocks) were 84% (UCLA), 78% (Harvard), 73% (Indiana), and 67% (Pitt), along with the percentage of cases with the presence of tumor tissue in 1 or more block per case. Correspondingly, Table 9 shows from the total blocks examined, the retrieval rate for the blocks with tumor present to be highest for GIST (80%) and mycosis fungoides (77%) followed by adrenal cortical carcinoma (68%) and adenoid cystic carcinoma (58%). UCLA and Indiana both (76%) presented with the greatest percentage of blocks with tumor found, followed by Harvard (70%) and Pittsburgh (61%).

**Figure 4:**
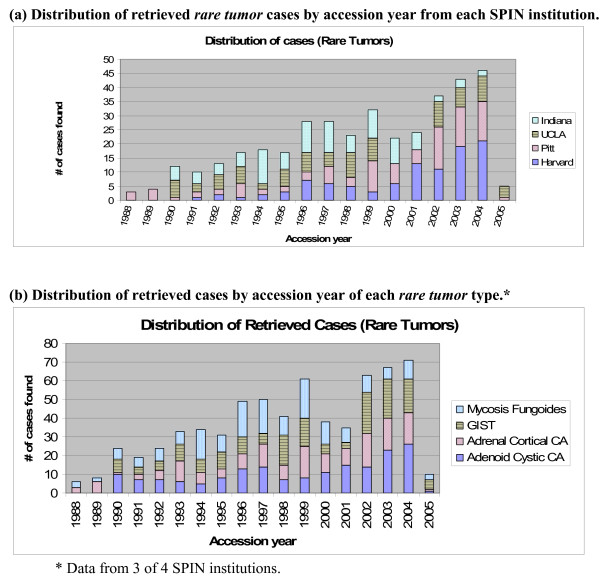
**Distribution of retrieved cases for *rare tumors***. Illustration of number of cases retrieved for rare tumor cases by the original year of accession for that case: (a) Breakdown by each institution and (b) by rare tumor type*. Specimens retrieved and evaluated for the rare tumors encompassed cases accessioned range from 1988–2005 with 62% being greater than 5 years old.

Although many of the cases did have paired blocks and slides with tumor available, Figure 2b shows that there were 11 cases that had slides with tumor available but no matching blocks, and 82 cases had neither the slides nor the blocks. The retrieval rate for tumor blocks from all centers for rare tumors was 66% with a mean largest tumor size of 1.56 cm, revealed in Table 10. The number of cases retrieved for the rare tumor types by the original year of accession is seen in Figure 4a and 4b, showing the distribution by the SPIN institutions and by the rare tumor types, respectively. Specimens retrieved and evaluated for the rare tumors encompassed cases accessioned from 1988–2005 with 62% greater than 5 years old.

## Discussion

Advances in proteomics and genomics technologies have led to a multitude of opportunities for research, the majority of which require high quality tissue specimens with associated annotation data[4,9,10]. Pathology reports and the clinical data contained within them are a valuable resource and an historically relatively underutilized method of obtaining the vast amount of tissue samples from existing paraffin archives for potential use in many of these research, educational and clinical projects [10-14]. With advances in information system technologies, more sophisticated resources have become available for data mining the rich textual information from archival pathology reports[4,15]. Although the importance of utilizing novel informatics techniques within pathology departments has been previously reported, there is little literature on the quality and availability of archived tissue collections[4]. As a precursor to a successful use of SPIN tools on a larger scale, we have analyzed a sample of pathology reports to determine the percentage of these reports for which we can find the associated tissue blocks with cancer that are still available for research purposes and which are retrievable from the warehouses.

Archived formalin-fixed paraffin-embedded tissue blocks are generally labeled with unique accession numbers and are remarkably resilient, which permits long-term storage and retrieval for research use. These resources are often managed locally by anatomic pathology laboratories and generate a wealth of material via routine diagnostic workups, particularly from resources associated with large academic centers which have larger collections and the expertise to report on rare tumors and classify new diagnostic markers for the common entities[4]. With the advent of advances in molecular biology tools, materials from archived paraffin blocks are amenable to extracting high quality biological material for use in proteomics and genomics projects[8]. Thus, if we can successfully identify blocks in significant numbers in quantity as well as quality for research use across multiple institutions, then implementation of tools like those developed by the SPIN could process electronic pathology reports that would dramatically increase the value of these collections.

In efforts to accelerate the pace of discovery for researching the genetic underpinnings of diseases, the National Cancer Institute (NCI) has sponsored several tissue annotation and banking efforts at the nationwide level[8,16-20]. However, many of these collections use labor-intensive manual processes to identify cases from archived tissue collections and legacy databases. The solution that the SPIN initiative proposes is to supply software tools that can be run via the internet at participating institutions in a HIPAA-compliant manner after agreeing with the bylaws of the consortium and approval of other members[3]. Currently, many of the SPIN members are intimately involved with enhancing many of the prototype SPIN tools created by this group by working with the Cancer Text Information Extraction System (CaTIES) project of the NCIs Cancer Bioinformatics Grid (caBIG) initiative. This relationship allows a common framework by which the SPIN institutions can integrate their data with the caBIG community.

In brief description, the mechanisms of how the SPIN tools function are best described by 4 major tasks performed prior to the data being searchable within a peer-to-peer model. First, scrubbing of electronic records by using a de-identification program would remove any HIPAA identifiers. Second, the reports are parsed into fields or chucks specified in the SPIN XML scheme. These fields are items such as clinical history, gross description, microscopic description, etc. Third, the text for each of these chunks is autocoded so that all the medical concepts contained in the text could receive a code derived from a standard unencumbered vocabulary. And finally, a strategy is devised to query this information by preserving the intended context of a report in the autocoded product (e.g. dealing with negatives, connecting organ sites with their appropriate modifiers or morphologies). Many of these functional and technical components that are utilized by the SPIN tools have been described elsewhere [21-27].

Because the number of available cases varied between institutions based on the expertise and patient population of a particular hospital, it was important for this study to examine the number of cases that could be retrieved from the four most common tumors and a sampling of rare tumor types at all of the SPIN institutions to normalize the specimen sampling. A correlation was seen with the expertise of the institute's pathology and surgical units in terms of locating more cases from those institutes that had a higher frequency of resections in the diagnosis of interest. An example of this was at Indiana University, which had a very large number of choriocarcinoma cases because of their expertise in the area of testicular tumors. Similarly, at institutes that focused on certain types of cancers for research only, we found that the attrition rates of blocks for those organs of interest was higher because many of their cases were being utilized by local investigators or sent out to outside investigators via collaborative projects. For example, the University of Pittsburgh has a large research focus on prostate cancer and it also participates in the Cooperative Prostate Cancer Tissue Resource (CPCTR) program[16,28] and therefore had higher attrition rates for blocks with prostate cancer tissue.

Using the local preferred methods for case identification, such as the routine use of the LIS or cancer registry tools, was undemanding because it utilized the existing workflows and personnel without involving the SPIN tool and personnel. However, this varied method did mark differences of how cases were initially identified and thus which cases were selected for the standardized pathology review. For example, one institution included outside referral cases only for rare tumor category but not the common tumors; other institutions excluded outside referral cases altogether during their case selection process. These assorted "routine" methods normally used at various institutions bring to surface many issues that still need to be addressed or resolved and are one of the key barriers for advancing translational research, the lack of quality biospecimens and its access. Compounding these issues and ultimately leading to delays in study completions are fundamental incompatibilities in the inter-institutional research guidelines and protocols. This highlights the importance of implementing informatics solutions that are standardized and have common tools, methods and vocabularies for identifying cases within a network of institutions that participate and agree to share their large archives of paraffin blocks.

Many investigators are also limited by the number of samples available for performing powerful statistical studies within their own institutions, especially for the rare tumor types. Thus, it is critical to develop and evaluate tools that are able to open up the vast available archives for sharing between institutions and provide the research community with efficacious information on these tools in order to increase the number of specimens that are available with relative ease of accessibility, but without ceding on the autonomy or control of each participating site[24,26,29]. Furthermore, even if the retrieval rates of this study hold constant, as shown in Tables 2, 3, 4 and 7, 8, 9, against the approximately 300 million specimens currently stored in pathology archives [6], the sheer number of cases that could be available for researchers demonstrates the necessity and value of developing such tools that the SPIN envisions. For example, if it is assumed that 1% (3 million) of all specimens banked were rare tumors, then the ability to retrieve 76%, or approximately 2.28 million specimens, of rare tumor cases signifies a rate of success that would be adequate to support research in a wide variety of experiments. Table 9 also reveals that 71% of all the blocks of rare tumors cases found represented blocks with tumor tissue present. It is also important to note that many of these specimens have multiple blocks associated with them, which further increases the number of individual blocks available for research.

Many studies will require comparison to normal controls. This study does not evaluate the availability of such controls but, given the high prevalence of non-cancer specimens in our collective archives, we believe that there will be several different sources of normal tissues. Of course the definition of "normal" will differ as a function of the questions asked in each study.

## Conclusion

Pre-existing archives of tissue blocks routinely saved in pathology departments are adequate sources of tissue blocks that can be used in many types of research efforts. The need for tools such as SPIN is indicated by the growing use of tissues, diagnostic specimens, and their related clinical data in biomedical research. Our results demonstrate that significant retrieval rates (all blocks), when measured by the total number of available blocks, are possible for acquiring both common (89%) and rare tumors (76%). And despite the various search methods utilized in this study to identify cases, this study does show the possibility of finding an adequate number of cases with paraffin blocks (of both tumor tissue as well as adjacent normal tissue) as far back as 1990 that could be useful for the research community. This assessment shows that individual institutions can utilize electronic data to search for archival tissues which are of interest to researchers. As a follow up to this study, we intend on solely using SPIN tools in order to estimate the resources required to use SPIN alone and to determine what sample yield that restriction generates.

## Competing interests

The author(s) declare that they have no competing interests.

## Authors' contributions

AAP and AVP contributed equally to the first draft of this manuscript. The Pittsburgh Team did the overall coordination and design of the study. DG, DS, EMH, UJB, TMU, SD, OS, and HY did the pathology review. MJB, ISK, JJB, and JRG were project liaisons from the SPIN Coordinating Committee. All authors reviewed and commented on successive drafts of the manuscript and approved the final manuscript to the first author.

## Pre-publication history

The pre-publication history for this paper can be accessed here:



## Figures and Tables

**Table 1:** Availability of slides/blocks from tissue archives for *common tumors*. Summary of the availability of slides and blocks retrieved from all SPIN sites with a breakdown of the number of blocks with presence of tumor for cases in the common tumor type category.

**Quantity**	**# of cases found on search: slides available by path report**	**# of cases found on search: slides found in archive**	**# of cases found on search: blocks available by path report**	**# of cases found on search: all blocks found in archive**	***# of cases found on search: Blocks w/Tumor found in archive***
n/a*	104	0	4	0	**97**
**0**	0	86	1	44	**10**
**1 – 3**	14	24	17	19	**115**
**4 – 6**	10	13	11	19	**106**
**7 – 9**	15	28	29	29	**48**
**10 – 14**	59	63	104	99	**22**
**15 – 19**	74	76	109	99	**2**
**20 – 29**	99	86	109	77	**0**
**30 – 39**	20	23	14	13	**0**
**>40**	5	1	2	1	**0**

	**400**	**400**	**400**	**400**	**400**

n/a * = not available, blocks only, no tumor seen

**Table 2:** Retrieval rates for *common tumors*. Retrieval rate from all SPIN sites for the common tumor type category: (a) Percentage case retrieval by each common tumor type (cases/total cases). Case retrieval rate for each tumor types in the *common tumors *category. (cases/total cases)

	**n = 100**	**n = 100**	**n = 100**	**n = 100**	**n = 400**
	
	**Breast**	**Colon**	**Lung**	**Prostate**	**ALL Common Cases**
**% case retrieval (all blocks)**	93%	85%	94%	84%	**89%**
**% case retrieval (Blocks with Tumor)**	74%	81%	84%	54%	**73%**

**Table 3:** Retrieval rates for *common tumors*. Retrieval rate from all SPIN sites for the common tumor type category: percentage case retrieval by each SPIN institution (cases/total cases); Case retrieval rate at each SPIN institution for *common tumors*. (cases/total cases)

	**n = 100**	**n = 100**	**n = 100**	**n = 100**
	
	**Pitt**	**UCLA**	**IU**	**Harvard**
**% case retrieval (all blocks)**	80%	98%	95%	83%
**% case retrieval (Blocks with Tumor)**	71%	94%	54%	74%

**Table 4:** Retrieval rates for *common tumors*. Retrieval rate from all SPIN sites for the common tumor type category: breakdown by each common tumor type at each institution (tumor blocks/all blocks). Retrieval rate for blocks with the presence of tumor for each *common tumor *type category at each SPIN institution. (tumor blocks/all blocks)

	**n = 25**	**n = 25**	**n = 25**	**n = 25**	**n = 100**
	
***SPIN INSTITUTIONS***	**Breast**	**Colon**	**Lung**	**Prostate**	**ALL Common Tumors**
**PITTSBURGH**	76%	96%	84%	84%	**85%**
**UCLA**	100%	92%	100%	100%	**98%**
**INDIANA**	88%	92%	100%	100%	**95%**
**HARVARD**	88%	64%	100%	80%	**83%**

**ALL SPIN SITES**	**88%**	**86%**	**96%**	**91%**	**90%**

**Table 5:** Average size of tumor present in tissue blocks. The average size of tumor present on the blocks for *common tumor *types retrieved and reported from a pool of 400 cases from all SPIN centers. *Common Tumors*

**Tumor Size**	**Largest block containing tumor (cm)**	**2nd largest block with tumor (cm)**	**3rd largest block with tumor (cm)**
***n***	293	258	211
**AVERAGE**	1.487	1.232	1.038
**MAX**	2.5	2.5	2.5
**MIN**	0.1	0.1	0.1
**STDEV**	0.753	0.765	0.756

**Table 6:** Availability of slides/blocks from tissue archives of *rare tumors*. Summary of the availability of slides and blocks retrieved from all SPIN sites with a breakdown of the number of blocks with presence of tumor for cases in the rare tumor type category.

**Quantity**	**# of cases found on search: slides available by path report**	**# of cases found on search: slides found in archive**	**# of cases found on search: blocks available by path report**	**# of cases found on search: all blocks found in archive**	***# of cases found on search: Blocks w/Tumor found in archive***
n/a*	114	18	42	18	**95**
**0**	0	71	16	80	**41**
**1 – 3**	78	81	114	109	**146**
**4 – 6**	34	43	50	44	**41**
**7 – 9**	21	25	34	30	**27**
**10 – 14**	37	56	58	49	**41**
**15 – 19**	45	35	42	38	**8**
**20 – 29**	42	43	30	23	**1**
**30 – 39**	17	18	12	8	**0**
**>40**	12	10	2	1	**0**

	**400**	**400**	**400**	**400**	**400**

n/a * = not available, blocks only, no tumor seen

**Table 7:** Retrieval rates for *rare tumors*. Retrieval rate from all SPIN sites for the rare tumor type category: Percentage case retrieval by each rare tumor type (cases/total cases). Case retrieval rate for each tumor types in the *rare tumors *category. (cases/total cases)

	**n = 100**	**n = 100**	**n = 100**	**n = 100**	**n = 400**
	
	**Adenoid Cystic Carcinoma**	**Adrenal Cortical Ca**	**GIST**	**Mycosis Fungoides**	**ALL Rare Cases**
**% case retrieval (all blocks)**	66%	71%	82%	83%	**76%**
**% case retrieval (Blocks with Tumor)**	58%	65%	72%	69%	**66%**

**Table 8:** Retrieval rates for *rare tumors*. Retrieval rate from all SPIN sites for the rare tumor type category: Percentage case retrieval by each SPIN institution (cases/total cases); Case retrieval rate at each SPIN institution for *rare tumors*. (cases/total cases)

	**n = 100**	**n = 100**	**n = 100**	**N = 100**
	
	**Pitt**	**UCLA**	**IU**	**Harvard**
**% case retrieval (all blocks)**	67%	84%	73%	78%
**% case retrieval (Blocks with Tumor)**	42%	76%	76%	70%

**Table 9:** Retrieval rates for *rare tumors*. Retrieval rate from all SPIN sites for the rare tumor type category: Breakdown by each rare tumor type at each institution (tumor blocks/all blocks). Retrieval rate for blocks with the presence of tumor for each *rare tumor *type category at each of the SPIN institution. (tumor blocks/all blocks)

	**n = 25**	**n = 25**	**n = 25**	**n = 25**	**n = 100**
	
***SPIN INSTITUTIONS***	**Adenoid Cystic Carcinoma**	**Adrenal Cortical Ca**	**GIST**	**Mycosis Fungoides**	**ALL Rare Cases**
**PITTSBURGH**	4%	96%	64%	80%	**61%**
**UCLA**	88%	36%	100%	80%	**76%**
**INDIANA**	68%	80%	80%	76%	**76%**
**HARVARD**	72%	60%	76%	72%	**70%**

**ALL SPIN SITES**	**58%**	**68%**	**80%**	**77%**	**71%**

**Table 10:** Average size of tumor present in tissue blocks. The average size of tumor present on the blocks for *rare tumor *types retrieved and reported from a pool of 400 cases from all SPIN centers. *Rare Tumors*

**Tumor Size**	**Largest block containing tumor (cm)**	**2nd largest block with tumor (cm)**	**3rd largest block with tumor (cm)**
***n***	286	221	167
**AVERAGE**	1.555	1.523	1.596^^^
**MAX**	2.6	2.7	2.8
**MIN**	0.1	0.1	0.1
**STDEV**	0.854	0.820	0.726

^^^The average size is based on all cases that reported the third block. Cases with *large volume *tumors had many blocks with large sizes of tumor (≥3 blocks), whereas, *small volume *tumors had fewer blocks with tumor available (< 3 blocks). The total number of the 3^rd ^block with the presence of tumor was many from *large volume *tumors, thus the higher average size compared to largest block #1.
